# Endoglin alleviates neuropathic pain by protecting the blood–spinal cord barrier through the TGF-β/Smad2 signaling pathway

**DOI:** 10.3389/fnmol.2025.1603619

**Published:** 2025-09-15

**Authors:** SiJing Liao, Fei-Ran Zhou, Qun Li, Tao Jin, Gui-Fang Xiang, Xin Wang, Yan Liu, Hq Zhu, Qing Liu, Yuexin Liu, Ying Zhang

**Affiliations:** ^1^Traditional Chinese Medicine Hospital of Meishan, Meishan, China; ^2^The Affiliated Hospital of Southwest Medical University, Luzhou, China; ^3^The Affiliated Traditional Chinese Medicine Hospital, Southwest Medical University, Luzhou, China; ^4^Suining First People’s Hospital, Suining, China; ^5^Hejiang Traditional Chinese Medicine Hospital, Luzhou, China; ^6^Zhongshan Hospital, Fudan University, Shanghai, China; ^7^Luzhou Key Laboratory of Research for Integrative on Pain and Perioperative Organ Protection, Luzhou, China

**Keywords:** endoglin, TGF-β/Smad2, neuropathic pain, blood–spinal cord barrier, endothelial cell

## Abstract

**Introduction:**

Central neuroinflammation is pivotal in neuropathic pain pathogenesis, with blood-spinal cord barrier (BSCB) dysfunction recognized as a trigger for neuroinflammation and pain, though molecular mechanisms remain poorly understood.

**Methods:**

Through comparative clinical studies measuring serum endoglin in postherpetic neuralgia (PHN) patients versus healthy controls, and animal investigations using spared nerve injury (SNI) rat models with recombinant endoglin intervention, we assessed mechanical/thermal hyperalgesia, microglial activation, inflammatory cytokines, BSCB permeability, and TGF-βRI/Smad2/NR2B phosphorylation.

**Results:**

PHN patients exhibited lower serum endoglin versus controls; SNI rats showed reduced spinal endoglin compared to sham controls. Recombinant endoglin alleviated hyperalgesia while reversing microglial activation, inflammation, BSCB impairment, and NR2B phosphorylation. SNI decreased spinal TGF-βRI expression and Smad2 phosphorylation.

**Discussion:**

These findings demonstrate that endoglin reduction disrupts BSCB integrity via TGF-β/Smad2 pathway inhibition in endothelial cells, driving microglial activation, neuroinflammation, and NR2B phosphorylation, thereby elucidating a key pain mechanism and identifying endoglin as a therapeutic target.

## Introduction

Neuropathic pain is widely recognized as one of the most challenging types of chronic pain to manage, with epidemiological studies indicating that it affects at least 30% of the global population (Cohen et al., 2021). Peripheral nerve injury (PNI) leading to neuropathic pain triggers multidimensional signaling pathway changes, one of the most prominent manifestations of which is neuroinflammation. Neuroinflammation is characterized by the infiltration of immune cells, glial cell activation, and the overexpression of neuroinflammatory cytokines (Basbaum et al., 2009). PNI can lead to structural destruction and increased permeability of BSCB, facilitating the migration of immune cells from circulation into the spinal parenchyma, the release of inflammatory mediators, and further activation of glial cells (Costigan et al., 2009). These central neuroinflammatory responses play a crucial role in both the initiation and maintenance of neuropathic pain (Zhang et al., 2007; Costigan et al., 2009; Tenorio et al., 2013; Xu et al., 2023). The BSCB is comprised of capillary endothelial cells, the basal lamina, pericytes, and astrocytes (Sauer et al., 2017). As a fundamental component of the spinal cord microenvironment, the BSCB serves to separate and connect the immune system with the peripheral and central nervous systems. However, the molecular mechanisms of BSCB dysfunction in neuropathic pain remain unclear.

Endothelial cells constitute a vital component of the blood–spinal cord barrier (BSCB) (Reinhold and Rittner, 2017). Endoglin, also known as CD105, is a type I transmembrane glycoprotein mainly expressed on the membranes of endothelial cells. It plays a crucial role in modulating the activity of endothelial cells in response to TGF-β signal transduction, influencing processes such as cell proliferation, differentiation, adhesion, and migration (Rakocevic et al., 2017). However, the role of endoglin in BSCB dysfunction and its regulation of neuropathic pain remains unknown.

Proteomics helps elucidate the pathogenesis of Neuropathic pain. Tandem mass tags (TMT) are an *in vitro* labeling technology that can be extensively applied to investigate differentially abundant proteins. Currently, the TMT-labeled quantitative proteomics technology can be combined with the highest-resolution liquid chromatography-tandem mass spectrometry (LC-MS/MS) technology and achieve an accurate qualitative and quantitative analysis of proteins in 2–18 different samples simultaneously. In this study, we employed TMT-labeled quantitative proteomic technology to identify differentially expressed proteins (DEPs) in the PHN group compared to the healthy group and found that patients with PHN had lower blood endoglin levels than healthy subjects. Based on this finding, subsequent experimental studies in a spared nerve injury (SNI) rat model were designed to explore the functional role and mechanistic involvement of endoglin in neuropathic pain pathogenesis, with particular focus on its potential interaction with the TGF-β signaling pathway and blood–spinal cord barrier (BSCB) function.

## Materials and methods

### Study participants

The trial was approved by the Human Subjects Ethics Committee at the Affiliated Traditional Chinese Medicine Hospital of Southwest Medical University (Approval No. KY2019054) and received authorization from the local ethics committee. It was also registered with the Chinese Clinical Trial Registry (Registration No. ChiCTR1900023655). All participants provided written informed consent upon inclusion and were recruited from the Affiliated Traditional Chinese Medicine Hospital of Southwest Medical University between June 2018 and April 2019. The trial adhered to the Declaration of Helsinki and Good Clinical Practice Guidelines.

Inclusion criteria were as follows: males and females aged between 50 and 80 years, with a body mass index (BMI) ranging from 18.0 to 25.0, a confirmed diagnosis of postherpetic neuralgia (PHN)—thoracic Herpes Zoster, and a visual analog scale (VAS) score of 4 or higher. Additionally, participants were required to have had inadequate pain relief from oral medications, including anticonvulsants, opioids, and antidepressants, for at least 1 month. Inclusion criteria for the healthy control group comprised: concurrently enrolled asymptomatic volunteers, age- and sex-matched to the PHN group, verified as healthy through medical examination with no significant organic or functional diseases; absence of other pain-related syndromes; no use of anticonvulsants, opioids, or antidepressants for pain relief within 1 month prior to enrollment; and provision of written informed consent.

Exclusion criteria included the presence of concomitant pain syndromes (e.g., complex regional pain syndrome, spinal cord injury, diabetic polyneuropathy, or fibromyalgia), serious concurrent systemic diseases (such as hyperlipidemia, hypertension, diabetes mellitus, myasthenia gravis, severe renal impairment, decreased lung function, shock, or liver dysfunction), infectious diseases (including human immunodeficiency virus (HIV), Hepatitis B, and syphilis), malignant tumors, and mental disorders (such as somatization, schizophrenia, or acute anxiety).

### Clinical samples

According to the inclusion and exclusion criteria, the participants were divided into two groups: the PHN group and the healthy group, and experiments were performed with three biological replicates for each group. Serum samples were collected from the aforementioned study participants upon enrollment.

### TMT-based quantitative proteomics analysis

Proteomic analysis of serum samples was performed by a TMT-based quantitative approach. The whole TMT-based quantitative proteomics analysis process includes protein extraction, sodium dodecyl sulfate-polyacrylamide gel electrophoresis (SDS-PAGE) electrophoresis, protein digestion, TMT labeling, reversed-phase liquid chromatography (RPLC) fractionation, liquid chromatography-tandem mass spectrometry (LC-MS/MS) analysis, and proteomics data analysis. Briefly, 40-μL of serum per sample was diluted with 10 × Binding Buffer and water. Albumin/IgG was removed using a spin column (pretreated with Binding Buffer). Samples were loaded, washed twice (600-μL Binding Buffer), and the filtrate was lyophilized. Proteins were resuspended in 300-μL of SDS lysis buffer, centrifuged (12,000 g, 15 min), and quantified via Bradford assay before storage at −80 °C. Then, 15-μg of protein per sample was separated on a 12% SDS-PAGE gel, fixed (2 h), and stained with Coomassie Brilliant Blue, Solarbio (C8430) (12 h). Gels were washed until bands were visible and scanned (300 dpi, GE ImageScanner, GE Healthcare). Notably, 100-μg of protein was reduced (10-mM of dithiothreitol (DTT), 60 °C, 1 h), alkylated (50-mM of iodoacetamide (IAA), 40 min, dark), and digested (trypsin, 37 °C, 12 h) on a 10 K ultrafiltration tube. Peptides were cleaned (100-mM of triethylammonium bicarbonate (TEAB), 12,000 rpm, 4 °C) and lyophilized. For TMT labeling, peptides were resuspended in 50-mM TEAB, mixed with TMT reagent (1 h, room temperature (RT)), and quenched with hydroxylamine. Labeled peptides were lyophilized and stored at −80 °C. Peptides were separated on an Agilent Zorbax reverse phase (RP, Agilent Zorbax) column (5 μm, 150 mm) using a gradient (2–98% acetonitrile (ACN), 300 μL/min, 210/280 nm). Fractions (8–50 min) were collected, lyophilized, and stored for mass spectrometry (MS). Samples were analyzed using a TripleTOF 5,600 MS (SCIEX, ThermoFisher (CHROMELEON7)) with a C18 trap column (3 cm × 100 μm) and analytical column (15 cm × 75 μm). Gradient: 2–95% ACN/0.1% formic acid (FA), 300 nL/min. MS settings: 2.4 kV spray voltage, 250-ms accumulation (400–1,500 m/z), and dynamic exclusion (22 s). MS/MS (100–1,500 m/z) was triggered for peaks with an intensity >260 (2–5 + charge).

### Data and bioinformatics analysis

ProteinPilot software (version 5.0) was used to search all of the TripleTOF 5,600 MS/MS raw data thoroughly against the sample protein database. Database searches were performed with Trypsin digestion specificity, and the cysteine alkylation was considered as a parameter in the database searching. For the protein quantification method, iTRAQ8-plex was selected. A global false discovery rate (FDR) of <1% was used, and peptide groups considered for quantification required at least two peptides. For differentially abundant proteins, Gene Ontology (GO) enrichment^1^ and Kyoto Encyclopedia of Genes and Genomes (KEGG) pathway^2^ analyses were performed to describe functions. The differences were identified as significant by Student’s *t*-test analysis (*p* < 0.05).

### Animal preparation and neuropathic pain model

Adult male Specific Pathogen Free (SPF) Sprague–Dawley (SD) rats, aged 6–8 weeks and weighing between 200 and 250 g, were obtained from the Animal Experimental Center of Southwest Medical University (SYXK (Sichuan) 2018–005). The animals were housed in pairs in a controlled environment at 23 ± 2 °C under a 12-h light/dark cycle, with ad libitum access to food and water. All experimental protocols were approved by the Laboratory Animal Ethics Committee of the Hospital of Traditional Chinese Medicine affiliated with Southwest Medical University (Approval No. 2020437).

Surgical procedures were performed under anesthesia using 2% isoflurane delivered in a mixture of 20% N_2_O and 80% O_2_. The right sciatic nerve was exposed at the trifurcation of the peroneal, tibial, and sural branches. The common peroneal and tibial nerves were ligated and transected, while the sural nerve was preserved intact. The sciatic nerve of rats with sham surgery was simply exposed without any further intervention (Tenorio et al., 2013). Following surgery, the rat’s surgical wounds were closed in layers: the fascia was approximated with 4-0 surgical sutures, followed by muscle repositioning and final skin closure. After suturing, povidone-iodine was applied for disinfection. Rats were monitored daily for signs of wound complications (swelling, redness, exudation, or dehiscence) following SNI surgery, with antibiotics administered if necessary.

### Drug administration

Intrathecal injections were performed via lumbar puncture to deliver the reagent into the cerebrospinal fluid, following a modified technique (Gao et al., 2009; Inoue and Tsuda, 2018; Lertkiatmongkol et al., 2016). Rats received daily intrathecal injections of either recombinant endoglin protein (2 μg/mL, 10 μL, ImmunoClone, United States) or saline (control) in the fifth to sixth lumbar vertebra (L5–L6), starting immediately after SNI and continuing until day 14. For knockdown of Smad2 in the spinal cord, rats received daily intrathecal injections of small interfering RNA (siRNA) (Smad2) (2 μg/mL, 10 μL) in L5–L6, starting immediately after SNI and continuing until day 14. The following sequences were chosen to generate siRNA: sense, 5′-TATAACATGTGAACCCTTT-3′; antisense, 5′TATAACATGTGGCAACCCTTT-3′. Smad2-siRNA was generated *in vitro* using a siRNA construction kit (Invitrogen, Carlsbad, CA, United States) according to the manufacturer’s instructions. A successful spinal puncture was confirmed by observing a brisk tail-flick response following needle entry into the subarachnoid space (Gao et al., 2009). The paw withdrawal threshold (PWT) and paw withdrawal latency (PWL) tests were conducted at 0, 3, 7, and 14 days post-SNI surgery.

### Assessment of thermal hyperalgesia and mechanical allodynia

The mechanical withdrawal threshold (MWT) was utilized to assess mechanical allodynia. For MWT measurements, rats were placed on a wire mesh floor inside a plastic box for 1 h to acclimatize to the testing environment. Von Frey filaments (North Coast Medical, San Jose, CA, United States), ranging from 2 to 26 g, were applied in ascending order to the plantar surface of the hind paw for approximately 5 s. The force that induced paw withdrawal was recorded. Each measurement was repeated across five sessions, with each session lasting no more than 5 s. After removing the extreme values, the average value was considered the MWT.

Thermal withdrawal latency (TWL) was employed to determine thermal hyperalgesia. For TWL measurements, each rat was placed in a box with a smooth, temperature-controlled glass floor. A heat source (Tes7370, Ugo Basile, Comerio, Italy) was focused on a section of the hind paw in contact with the glass, delivering a radiant thermal stimulus. Care was taken to monitor the heat application to prevent thermal damage to the plantar surface. The withdrawal latency of the right hind limb was recorded during thermal exposure. Each measurement was repeated across five sessions, allowing a 5-s interval between sessions. The hind paws were assessed alternately during consecutive tests, with intervals of at least 3 min between assessments. The TWL of the rats was calculated by averaging the values after excluding extreme measurements. To reduce the variability within individual rats in behavioral tests, we acclimatized the rats for 6 days before behavioral experiments.

### Enzyme-linked immunosorbent assay (ELISA)

Blood was collected immediately by cardiac puncture into prechilled tubes containing EDTA under 2% isoflurane anesthesia. The blood samples of both humans and rats were allowed to clot for 2 h at room temperature, then centrifuged at 8,000 rpm for 15 min at 4 °C, and the supernatant serum (plasma) was collected and subsequently stored at −80 °C until analysis. The inflammatory tissues from the L4–L6 spinal cord were extracted, snap-frozen in liquid nitrogen, homogenized, and transferred to 1.5-mL electropolished (EP) tubes stored at −80 °C until analysis. The levels of endoglin, interleukin 1β (IL-1β), tumor necrosis factor alpha (TNF-α), and IL-6 in the serum and tissues were measured using commercial enzyme-linked immunosorbent assay (ELISA) kits (Shanghai Beyotime Biotechnology, IL-6:PI328;IL-1β:PI303:TNF-α:PT516) according to standard procedures.

### Transmission electron microscope

Transmission electron microscope (TEM) was employed to examine the integrity of the blood–spinal cord barrier in rats. Tissue samples were prefixed with 3% glutaraldehyde, followed by post-fixation in 1% osmium tetroxide. Subsequently, tissues underwent dehydration through a series of acetone treatments, were infiltrated with Epox 812, EMS (14120) for an extended period, and then embedded. Semithin sections were stained with methylene blue, while ultrathin sections were prepared using a diamond knife and stained with uranyl acetate and lead citrate. The sections were analyzed using a JEM-1400-FLASH transmission electron microscope, JEM-1400FLASH (Shasha et al., 2022).

### Evans blue assay

The integrity of the BSCB was evaluated utilizing the Evans Blue (EB) leakage assay. The Evans Blue (EB) assay was performed as previously described (Goldim et al., 2019). Briefly, rats were anesthetized with 2% isoflurane and injected with EB (2 mL/kg). All injections were timed so that animals were exposed to the EB dye for 1 h when their mechanical thresholds were found to be lowest. Following this, animals were transcardially perfused with saline, and L4-L6 spinal cords were collected. Tissue weights were measured, and 1 mL of 50% (w/v in saline) trichloroacetic acid (TCA) was added per gram of tissue. Samples were homogenized and centrifuged for 20 min at 10,000 rpm. Supernatants were collected, diluted 4 times with ethanol, and the concentration of the extracted Evans Blue dye was determined spectrophotometrically at 620 nm, normalizing the mean dye content to the weight of the dissected spinal cord regions.

### Immunofluorescence

Rats were anesthetized with 2% isoflurane and perfused through the ascending aorta with phosphate buffered saline (PBS), followed by 4% paraformaldehyde. Following perfusion, the L4–L6 spinal cord segments were removed and post-fixed in 4% paraformaldehyde overnight. Spinal cord segments were sectioned into 10-μm slices using a freezing microtome. Following a blocking step with 10% goat serum containing 0.3% Triton X-100 (Abcam), ab286840 for 1 h at 37 °C, slices were incubated overnight at 4 °C with anti-Iba-1 antibody (rabbit, 1:500, Abcam, ab178846) diluted in 10% goat serum. After three rinses with phosphate-buffered saline (PBS) for 10 min each, slices were incubated for 1 h at 37 °C with a fluorescein isothiocyanate (FITC)-conjugated secondary antibody (1:1,000, Abcam, ab6785). Subsequently, slices were washed three additional times and counterstained with a mounting medium antifade with 4′,6-diamidino-2-phenylindole (DAPI) (Solarbio, Beijing, China) for 5 min. Images were captured using a fluorescence microscope (Leica, Germany).

### Western blotting

Western blotting analysis was employed to quantify changes in protein expression. Rats were anesthetized with 2% isoflurane and perfused with phosphate-buffered saline (PBS) at 4 °C. The L4–L6 spinal cord was rapidly extracted and homogenized in ice-cold RIPA lysis buffer (Beyotime Corporation, Shanghai, China) following the manufacturer’s instructions. The lysates were stored at −80 °C until further analysis. Protein concentrations were measured using a bicinchoninic acid (BCA) Colorimetric Assay Kit (Thermo Scientific, 23227). Subsequently, spinal cord protein samples were loaded onto SDS-polyacrylamide gels and subjected to electrophoresis at 80 V for 90 min. Proteins were then transferred to a 0.2 μm nitrocellulose membrane using an electrical transfer process for 1 h; the membranes were blocked with 5% non-fat milk for 2 h at room temperature (Bio-Rad, United States). Following blocking, membranes were incubated overnight at 4 °C with various primary antibodies: anti-endoglin (mouse, 1:200, Santa Cruz, CA, United States), anti-TGF-βRI (rabbit, 1:1,000, Thermo Fisher Scientific, MA1-21595), anti-Smad2 (rabbit, 1:1,000, CST, 5339S), anti-p-Smad2 (rabbit, 1:1,000, CST, 8828S), and anti-β-actin monoclonal antibody (rabbit, 1:5000, Abcam, ab6276). After three rounds of washing with TBST for 10 min each, membranes were incubated at room temperature for 2 h with a horseradish peroxidase (HRP)-conjugated goat anti-rabbit secondary antibody (1:5,000, Beyotime, Shanghai, China). Membranes were treated with Western Chemiluminescent HRP Substrate (Biosharp, BL523B, BL520B) for 5 min in the dark. Resulting images were quantified and analyzed using ImageJ software (National Institutes of Health, MD, United States), with protein levels normalized to those of β-actin.

### Statistical analysis

Data are expressed as mean ± standard error of the mean (SEM) and were analyzed by investigators blinded to the experimental design. The unpaired Student’s *t*-test (two-tailed), one-way analysis of variance (ANOVA) followed by Tukey’s multiple comparisons test, and one-way and two-way repeated-measures ANOVA followed by Tukey’s multiple comparisons test were used. A criterion *α* level of 0.05 was set.

## Results

### Endoglin level was decreased in the peripheral blood serum of PHN patients

**Figure 1:**
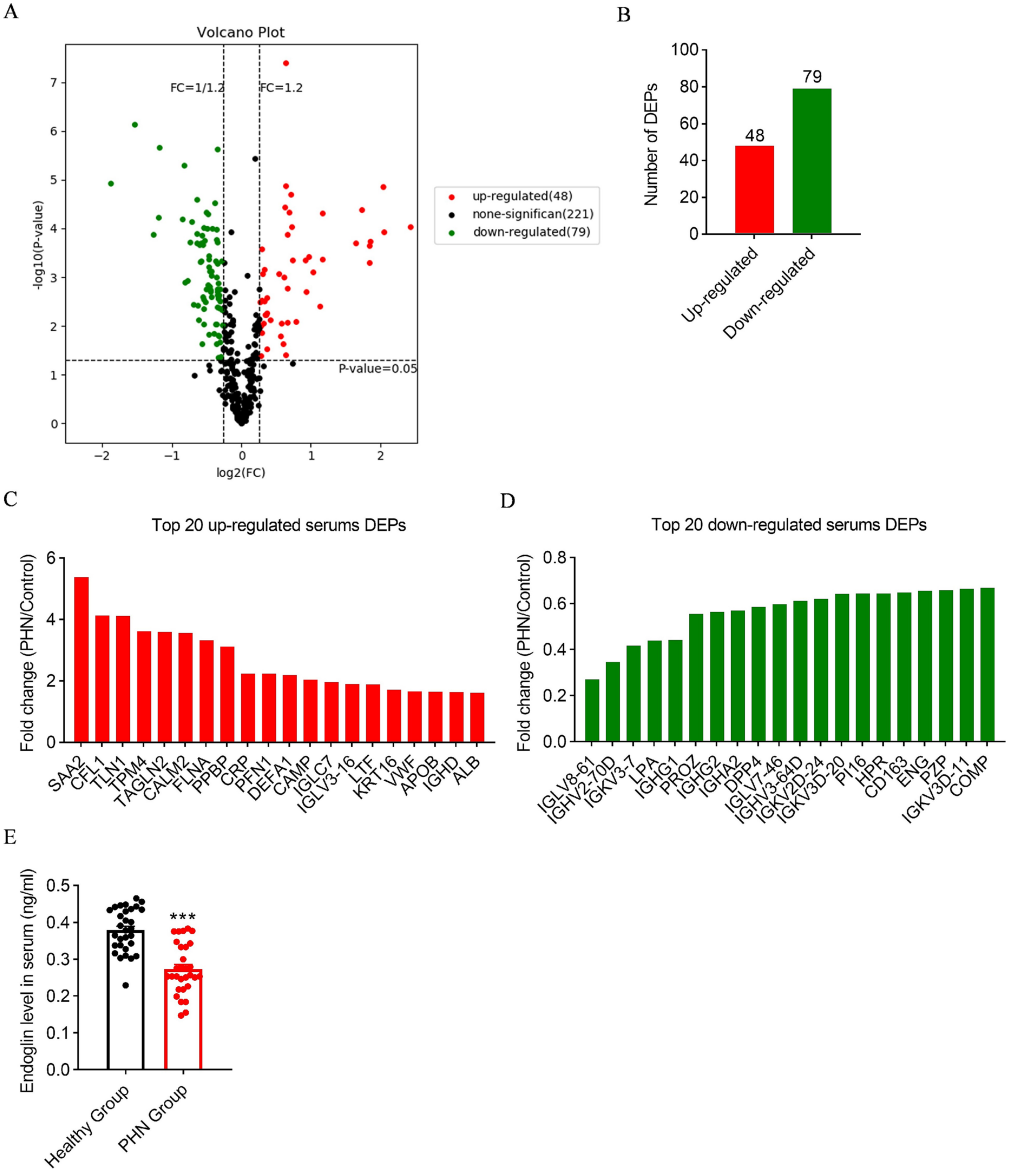
Endoglin expression decreased in the peripheral blood of PHN patients. **(A)** Protein microarray showed the differential expression of proteins in the peripheral blood of PHN patients and healthy subjects; *n* = 3. **(B)** The results showed that 48 proteins were upregulated and 79 proteins were downregulated. **(C)** The top 20 upregulated proteins. **(D)** The top 20 downregulated proteins. **(E)** ELISA analysis showed endoglin was significantly downregulated in the serum of PHN patients compared with healthy subjects. PHN, postherpetic neuralgia; *n* = 30 participants in each group. Significance was assessed by a two-tailed unpaired Student’s *t*-test **(E)**. ^***^p < 0.001. Data were presented as mean ± SEM.

To identify differentially expressed proteins in the peripheral blood of PHN patients compared to healthy subjects, we first performed a screening using protein microarray technology (Figure 1A). The results showed that 48 proteins were upregulated while 79 proteins were downregulated (Figure 1B). Notably, endoglin was identified as the fourth most downregulated protein among these 79 proteins (Figures 1C,D).

Next, we recruited 30 healthy volunteers and 30 PHN patients and measured their peripheral blood endoglin levels to further validate the differential expression of endoglin in PHN patients. The results showed that the peripheral blood endoglin levels of PHN patients were significantly lower than those of healthy volunteers (Figure 1E). In addition, the visual analog scale (VAS) scores of PHN patients were significantly higher than those of healthy subjects, but no significant differences in age, gender or body mass index (BMI) were observed between the two groups (Table 1).

**Table 1:** The characteristics and VAS score of health and PHN groups.

Group	Number	Age (years)	Gender (male/female)	BMI (kg/m^2^)	VAS (scores)	Endoglin (ng/mL)
Health group	30	66.7 ± 8.9	13/17	20.3 ± 1.6	0	79.03 ± 16.23
PHN group	30	63.9 ± 8.8	19/11	21.4 ± 1.2	6.0 ± 0.8	66.74 ± 19.58
*t*/*x*^2^	/	1.16	0.534	1.05	/	3.02
*p*	/	0.25	0.58	0.32	/	0.00

### Downregulation of endoglin level in peripheral blood serum and spinal cord of SNI rats

**Figure 2:**
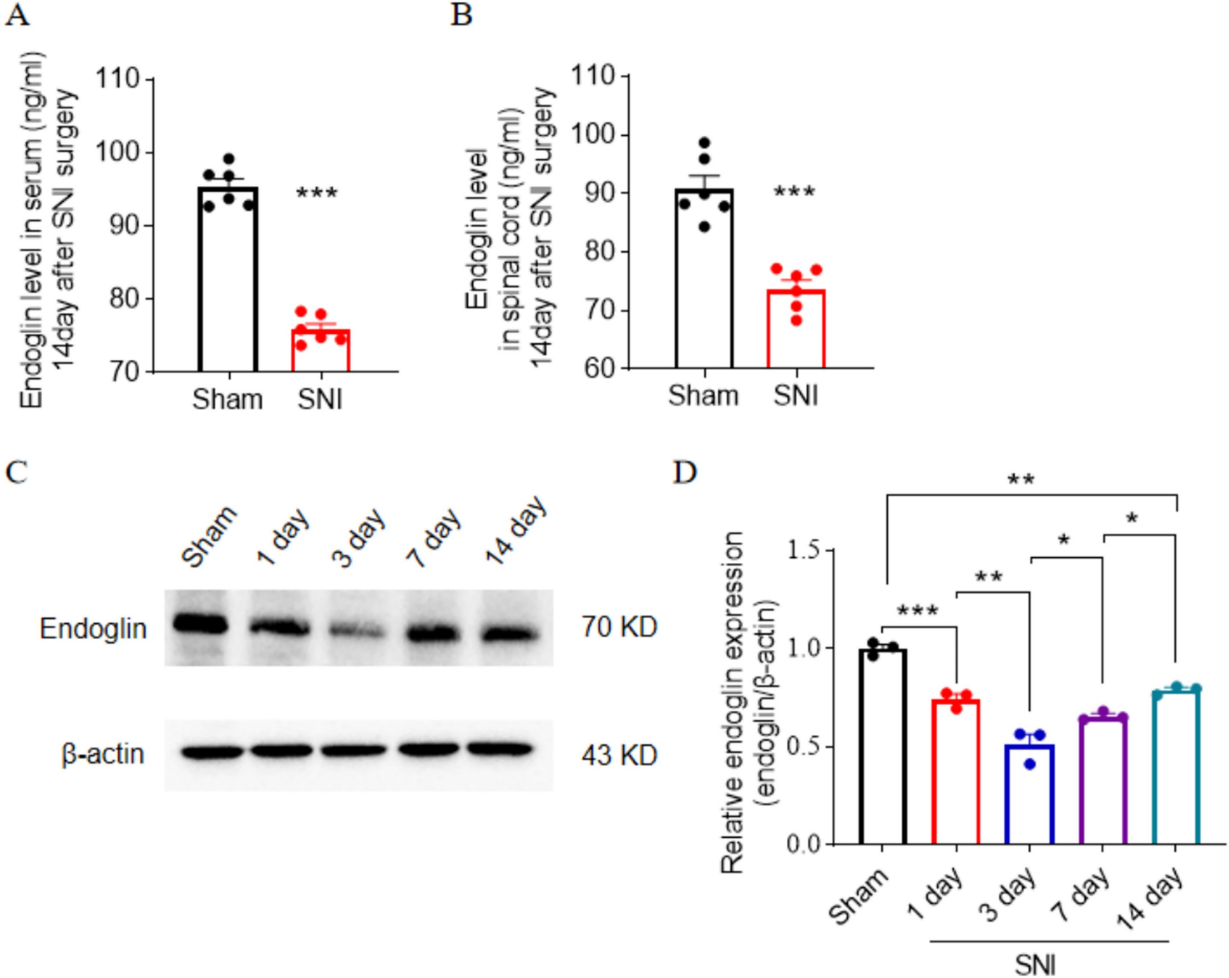
Endoglin expression was downregulated in the peripheral blood and spinal cord of SNI rats. **(A,B)** ELISA showed that the levels of endoglin were significantly downregulated in the peripheral blood **(A)** and spinal cord **(B)** of SNI rats compared with sham rats at 14 days after SNI; *n* = 6 rats in each group. **(C,D)** Time course of expression of endoglin in the spinal cord of rats after SNI. **(C)** Representative immunoblots. **(D)** Data summary; *n* = 3 rats for each group. Significance was assessed by two-tailed unpaired Student’s *t*-test **(A,B)** and one-way ANOVA followed by Tukey’s multiple comparisons test **(D)**. ^*^p < 0.05, ^**^p < 0.01, and ^***^p < 0.001. Data were presented as mean ± SEM.

We then examined the expression of endoglin in the peripheral blood of rats subjected to spinal nerve injury (SNI), which serves as an animal model for neuropathic pain. ELISA results showed SNI intervention significantly inhibited endoglin expression, consistent with our findings in patients with postherpetic neuralgia (PHN) (Figure 2A). To further investigate whether differential expression of endoglin occurs in the spinal cord at a critical time point in the maintenance of chronic pain, we assessed endoglin levels in the spinal cords of both the SNI and sham groups by ELISA and found that SNI intervention significantly reduced endoglin concentration in the L4–L6 spinal cord at 14 days after SNI (Figure 2B). Additionally, we utilized the Western blot to assess the expression of endoglin in the spinal cords of rats at 1, 3, 7, and 14 days following SNI. Our findings indicated that the expression of endoglin was significantly downregulated at 1, 3, 7, and 14 days following SNI. Notably, the expression of endoglin reached its nadir at 3 days after SNI (Figures 2C,D).

### Intrathecal injection of recombinant endoglin protein significantly alleviates hyperalgesia in SNI rats

**Figure 3:**
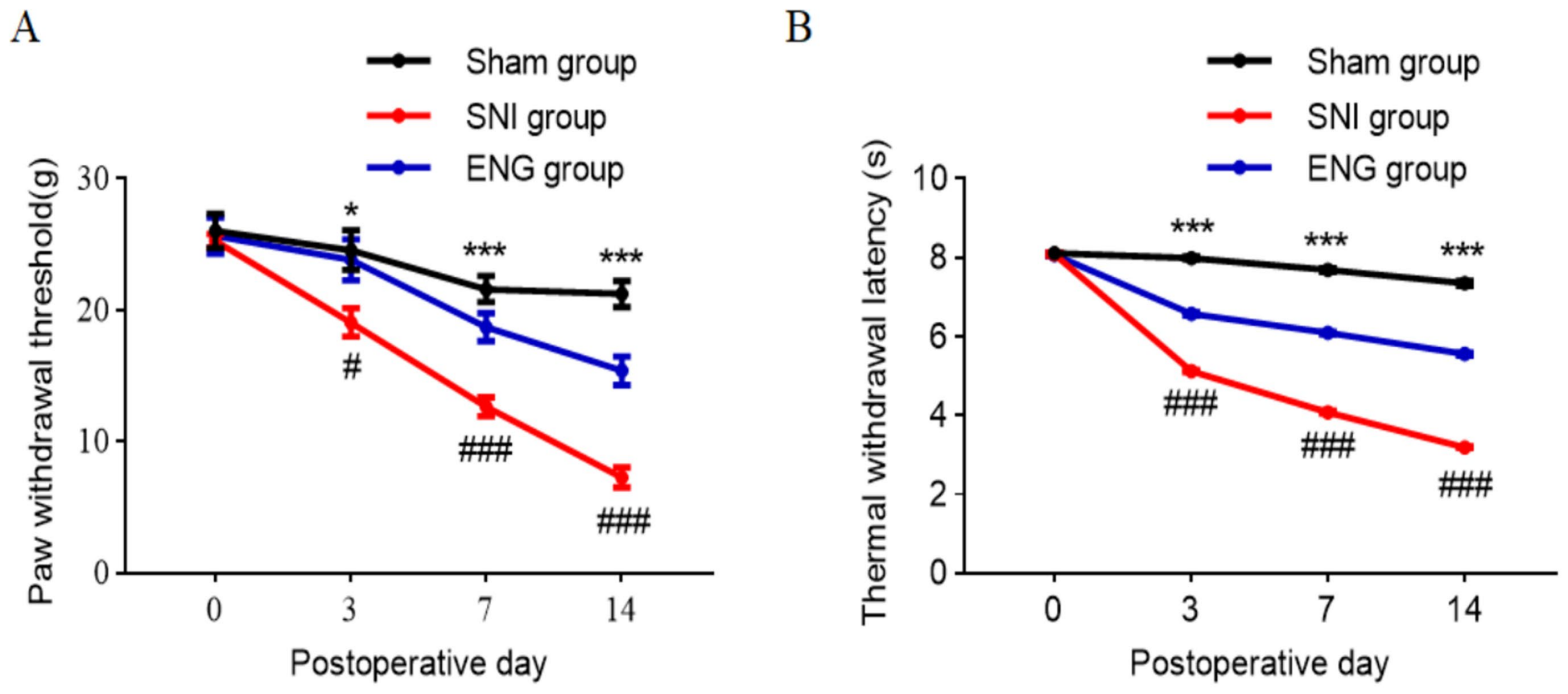
Intrathecal injection of recombinant endoglin protein significantly alleviates hyperalgesia in SNI rats. **(A)** The paw withdrawal threshold of all rats on the day of SNI and at 3, 7, and 14 days after SNI; *n* = 30 rats in each group. **(B)** The thermal withdrawal latency of all rats on the day of SNI and at 3, 7, 14 days after SNI; *n* = 30 rats in each group. Significance was assessed by two-way repeated-measures ANOVA followed by Tukey’s multiple comparisons test **(A,B)**. ^*^p < 0.05, ^**^p < 0.01, and ^***^p < 0.001. Data were presented as mean ± SEM.

To examine the effect of spinal endoglin on neuropathic pain, recombinant endoglin protein (2 μg/mL, 10 μL) was intrathecally administered to SNI rats once a day. Rats that received SNI exhibited mechanical and thermal hyperalgesia. Conversely, Intrathecal injection of recombinant endoglin protein significantly alleviate hyperalgesia induced by SNI (Figures 3A,B). These findings suggest that endoglin in spinal cord may play an important role in development and persistence of neuropathic pain.

### Intrathecal injection of recombinant endoglin protein can inhibit microglial activation and reduce inflammation in the spinal cord

**Figure 4:**
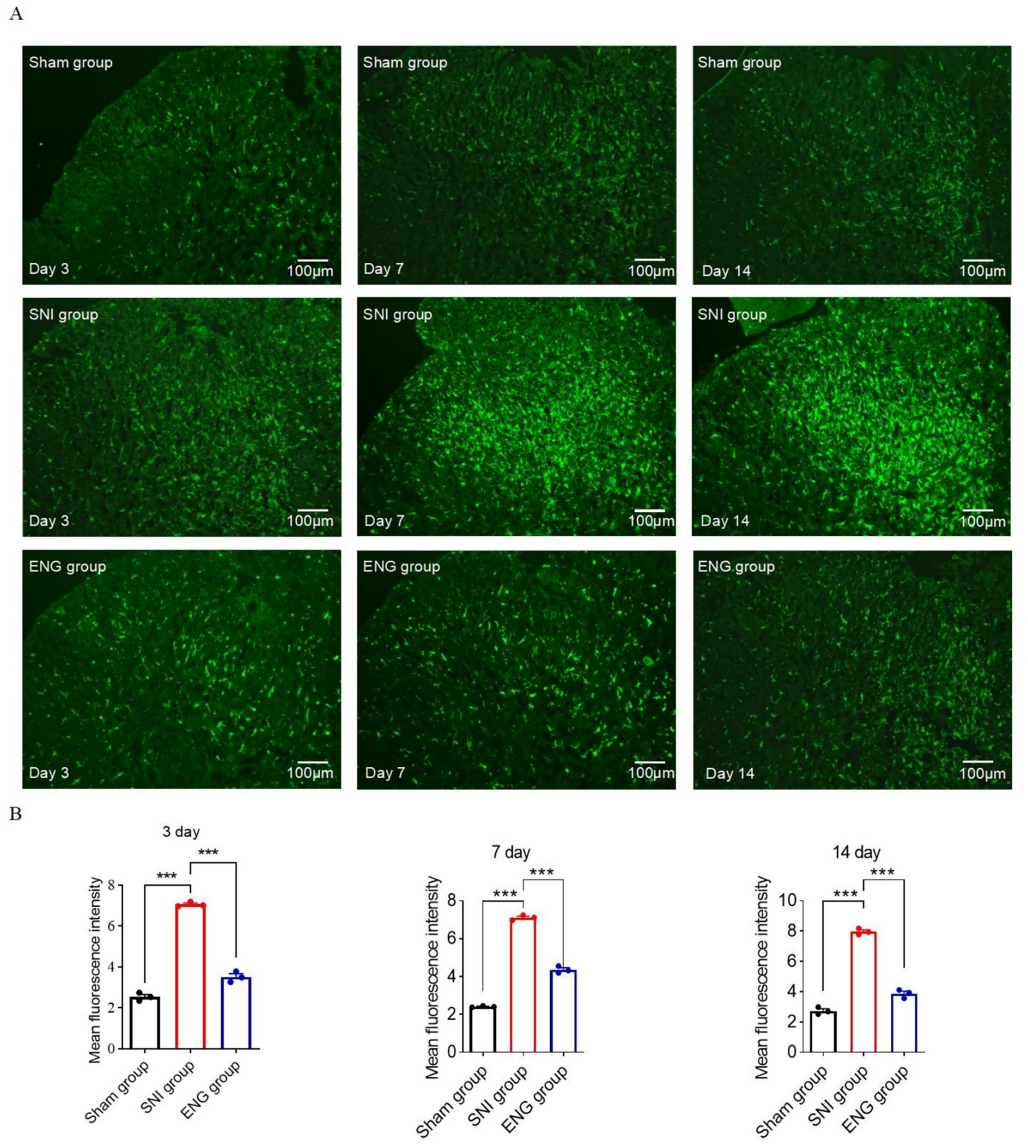
Intrathecal injection of recombinant endoglin protein significantly inhibited the microglia cell activation and neuroinflammation at L4–L6 spinal dorsal horn. **(A)** The represent immunofluorescence staining of Iba-1 (green) in the spinal dorsal horn of three groups of rats at 3, 7, and 14 days after SNI. **(B)** Data summary further confirmed that intrathecal injection of recombinant endoglin protein significantly inhibited the microglia cell activation; *n* = 3 rats in each group. Significance was assessed by one-way ANOVA followed by Tukey’s multiple comparisons test **(B)**. ^***^p < 0.001. Data were presented as mean ± SEM.

Activation of microglia in the spinal cord plays a critical role in the manifestation of neuropathic pain (Inoue and Tsuda, 2018). To investigate the effect of endoglin on microglia of the spinal cord, we performed Iba-1 immunostaining to assess the activation of microglia in the L4–L6 region. The results showed that activation of microglia occurs at 3, 7, and 14 days after SNI, which was reversed by intrathecal injection of recombinant endoglin protein (Figures 4A,B).

**Figure 5:**
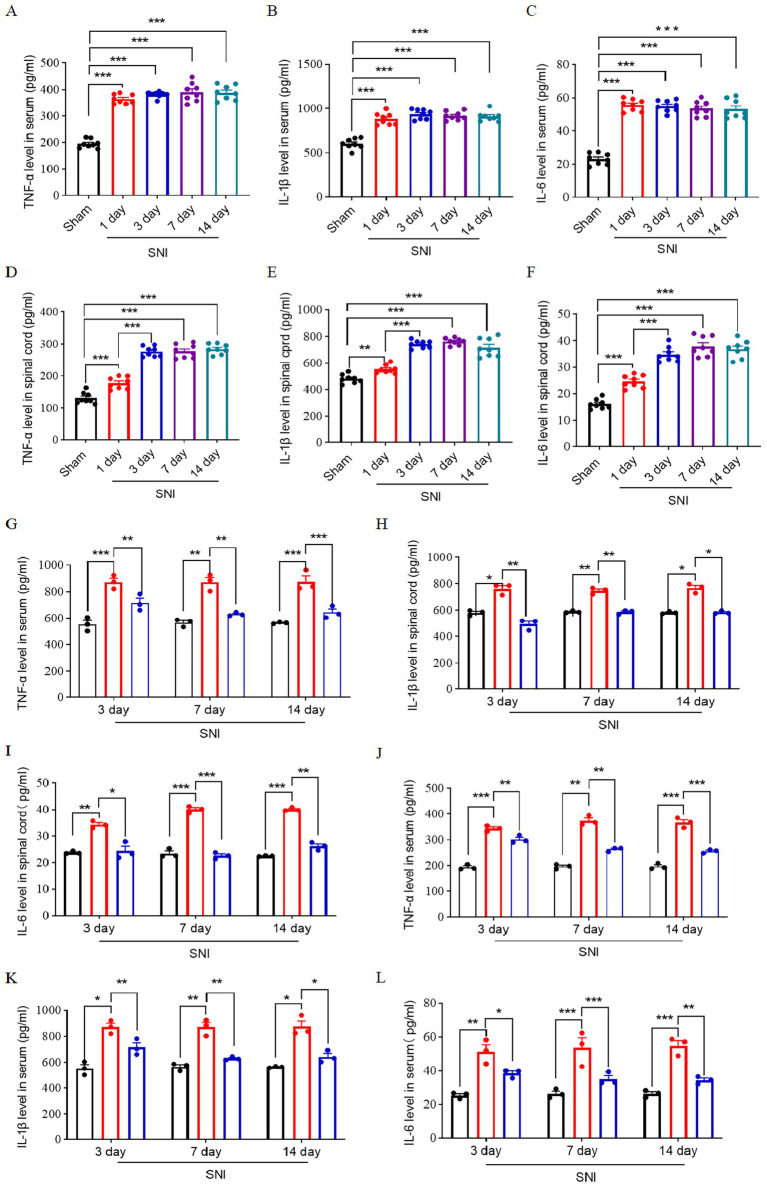
Expression of inflammatory factors in the peripheral blood and spinal cord of SNI rats. Time course of expression of TNF-α **(A)**, IL-1β **(B)**, and IL-6 **(C)** in peripheral blood serum; *n* = 8. Time course of expression of TNF-α **(D)**, IL-1β **(E)**, IL-6 **(F)** in spinal cord L4–L6 of rats; *n* = 8. The ELISA result shows the expression level of TNF-α **(G)**, IL-1β **(H)**, and IL-6 **(I)** in L4–L6 spinal dorsal horn at 3, 7, and 14 days after SNI in three groups. The ELISA result shows the expression level of TNF-α **(G)**, IL-1β **(H)**, and IL-6 **(I)** in peripheral blood serum at 3, 7, and 14 days after SNI in three groups. The black histogram represents the Sham group, the red histogram represents the SNI group, and the blue histogram represents the ENG group; *n* = 3 rats in each group. Significance was assessed by one-way ANOVA followed by Tukey’s multiple comparisons test **(A–F)** and two-way repeated-measures ANOVA followed by Tukey’s multiple comparisons test **(G–I)**. ^*^p < 0.05, ^**^p < 0.01, and ^***^p < 0.001. Data were presented as mean ± SEM.

Central inflammation plays a critical role in the development of neuropathic pain, with the onset of central inflammation frequently linked to the infiltration of peripheral immune cells and factors that breach the blood–spinal cord barrier (Jin et al., 2021). We quantify the levels of inflammatory cytokines TNF-α, IL-1β, and IL-6 in peripheral blood serum and the L4–L6 spinal cord in a time-course manner. The findings indicated that the levels of peripheral inflammatory cytokines increased and reached a peak at 1 day after SNI. These cytokines in the spinal cord increased and peaked at 3 days after SNI and remained until 14 days after SNI (Figures 5A–F). This observed temporal pattern, characterized by an initial peak in peripheral inflammatory factors followed by a subsequent peak in spinal cord inflammatory factors, suggests a potential correlation between central inflammation and peripheral inflammatory infiltration.

Then, we investigate the effect of endoglin on inflammatory cytokines of the spinal cord and peripheral blood. We found that the expression of these inflammatory factors was significantly elevated in the peripheral blood and L4–L6 spinal cord of SNI rats, while treatment with recombinant endoglin protein suppressed this upregulation (Figures 5G–L). These findings suggest that a reduction in endoglin may contribute to the onset and progression of neuropathic pain by modulating neuroinflammatory processes and microglia activation, and that recombinant endoglin protein may exert a protective effect by modulating these pathways.

### Intrathecal injection of recombinant endoglin protein alleviates BSCB dysfunction induced by SNI

**Figure 6:**
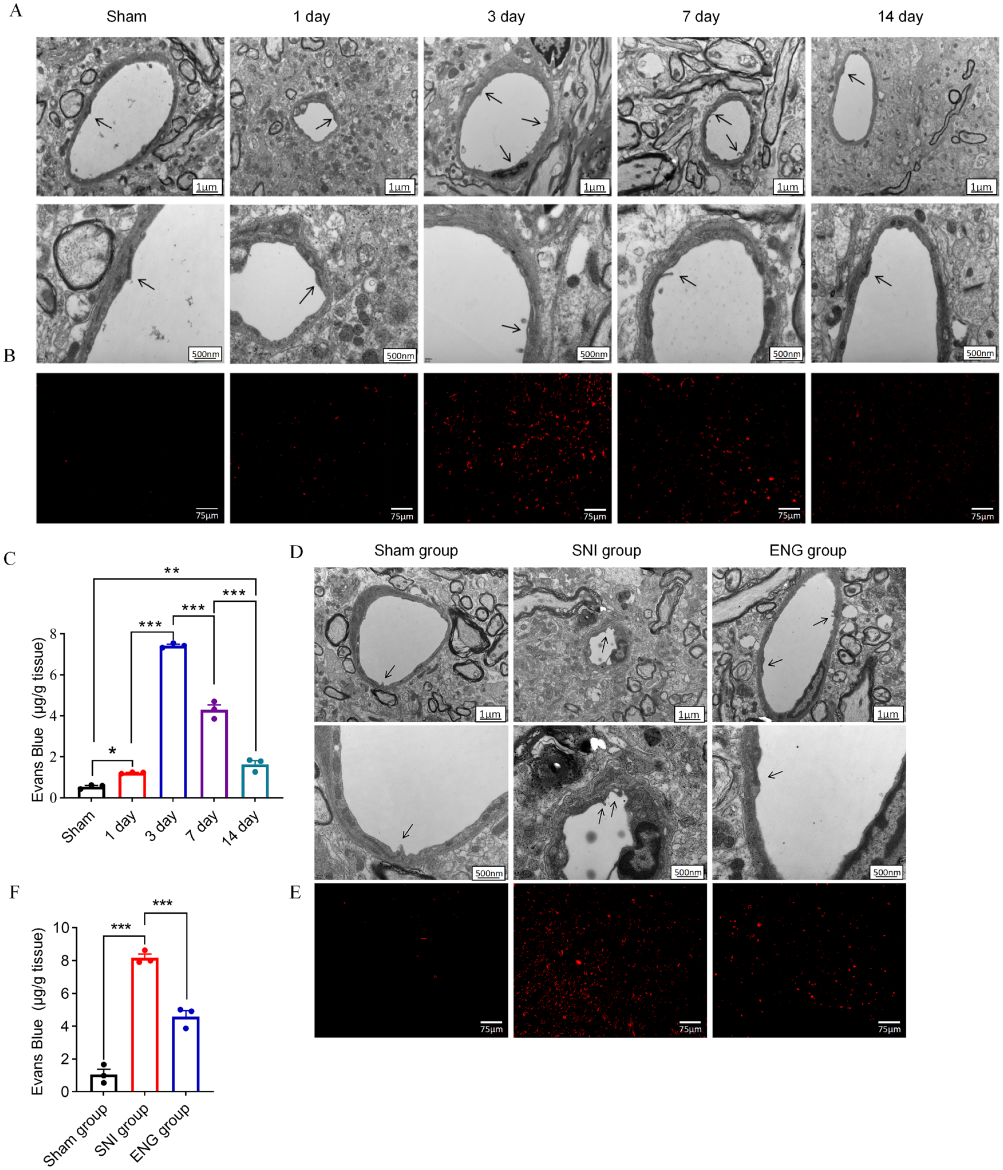
Dynamic changes in the structure and permeability of the blood–spinal cord barrier. **(A)** Dynamic structure changes of the blood–spinal barrier after SNI. The black arrow indicates the tight junction of endothelial cells. **(B)** changes of blood–spinal barrier permeability after SNI staining. **(C)** Quantitative analysis of Evens Blue in the L4–L6 spinal cord of SNI rats at each time point; *n* = 3 rats in each group. **(D)** The blood–spinal barrier structure of rats in each group. The black arrow indicates the tight junction of endothelial cells. **(E)** Blood–spinal barrier permeability of rats in each group. **(F)** Quantitative analysis of Evens Blue in the L4–L6 spinal cord of three groups; *n* = 3 rats in each group. Significance was assessed by one-way ANOVA followed by Tukey’s multiple comparisons test **(C,F)**. ^*^p < 0.05, ^**^p < 0.01, and ^***^p < 0.001. Data were presented as mean ± SEM.

The impairment of the blood–spinal cord barrier contributed to peripheral inflammatory infiltration (Montague-Cardoso and Malcangio, 2021). To investigate whether the damage of blood–spinal cord barrier occurs in neuropathic pain, we employed transmission electron microscopy and Evans Blue staining to assess the dynamic alterations in the structure and function of the blood–spinal cord barrier in L4–L6 of rats induced by SNI. The results showed that relative to the rats without SNI, a significant structural disruption of the blood–spinal cord barrier occurred at 3 days after SNI, but not 1 day after SNI, manifested as irregular endothelial cell surface and loosely connected endothelial cells. At 7 days after SNI, loosely connected endothelial cells remained, while the endothelial cell surface was smooth. At 14 days after SNI, structural disruption in the blood–spinal cord barrier was reversed, manifested as regular endothelial cell surface and tightly connected endothelial cells. These electron microscopy results suggest that the most pronounced structural disruption occurred at 3 days after SNI (Figure 6A).

Additionally, the results from the Evans Blue staining experiments showed that, in comparison to the rats without SNI, leakage of Evans Blue dye was evident at 1, 3, 7, and 14 days after SNI, with the most significant leakage occurring at 3 days after SNI, indicating a substantial increase in the permeability of the blood–spinal cord barrier (Figures 6B,C). Collectively, these findings suggest that the structural and functional integrity of the blood–spinal cord barrier in the dorsal horn of the L4–L6 spinal cord in rats is compromised following SNI, with the most severe structural damage and highest permeability observed at 3 days after SNI, which is consistent with the expression pattern of endoglin.

To examine the role of endoglin on the structure and function of the blood–spinal cord barrier within the dorsal horn of SNI rats, we intrathecally injected recombinant endoglin protein (2 μg/mL, 10 μL) into SNI rats once a day, then conducted transmission electron microscopy and Evans Blue staining at 3 days after SNI. The findings showed that the SNI group exhibited marked structural disruption of the blood–spinal cord barrier, characterized by pronounced gaps in the tight junctions of endothelial cells. Conversely, following intrathecal administration of recombinant endoglin protein reversed SNI-induced structural disruption and increased permeability of the blood–spinal cord barrier (Figures 6D–F). These findings support that downregulated endoglin facilitates structural and functional damage of the blood–spinal cord barrier in the dorsal horn of SNI rats.

### Intrathecal injection of recombinant endoglin protein inhibits the phosphorylation of NR2B at Tyr1472 induced by SNI

**Figure 7:**
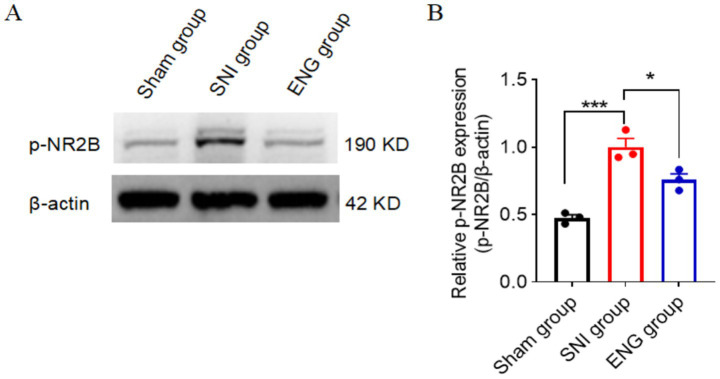
Intrathecal injection of recombinant endoglin protein inhibits SNI-induced phosphorylation of NR2B at Tyr1472. Phosphorylation level of Tyr1472-NR2B in the spinal cord at 14 days after SNI. **(A)** Representative immunoblots. **(B)** Data summary (*n* = 3 rats for each group). Significance was assessed by one-way ANOVA followed by Tukey’s multiple comparisons test. ^*^p < 0.05, ^**^p < 0.01, and ^***^p < 0.001. Data were presented as mean ± SEM.

The activation of N-methyl-D-aspartate receptors (NMDAR) in the spinal cord has been implicated in the development and maintenance of chronic neuropathic pain (Peterson et al., 2021). NR2B, a regulatory subunit of NMDARs, plays a critical role in this process. Phosphorylation of NR2B at Tyr1472 stabilizes NMDARs on the plasma membrane and enhances NMDA receptor-mediated calcium influx, thereby contributing to neuronal hyperexcitability and pain sensitization (Chen and Roche, 2007). Previous studies have demonstrated that the development and maintenance of neuropathic pain following peripheral nerve injury is critically dependent on phosphorylation of Tyr1472-NR2B in spinal dorsal horn neurons (Katano et al., 2016). To investigate the role of endoglin in this process, we examined its effect on phosphorylation of Tyr1472-NR2B. Our results revealed that phosphorylation of Tyr1472-NR2B was significantly increased in the L4–L6 spinal cord at 14 days after SNI. However, intrathecal administration of recombinant endoglin protein effectively suppressed this phosphorylation (Figures 7A,B). These findings suggest that a reduction in endoglin levels contributes to NMDAR activation by promoting phosphorylation of Tyr1472-NR2B, and that recombinant endoglin protein may exert a protective effect by modulating this pathway.

### Intrathecal injection of recombinant endoglin protein inhibits SNI-induced BSCB dysfunction through the TGF-β/Smad2 signaling pathway

**Figure 8:**
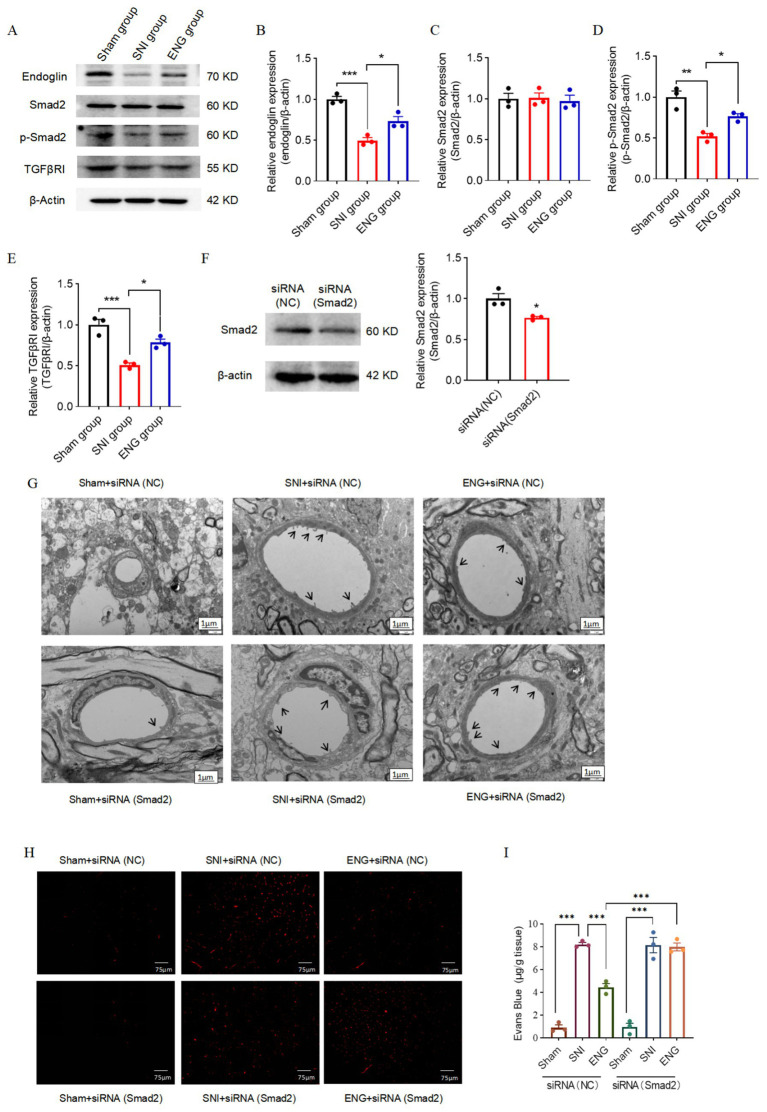
Intrathecal injection of recombinant endoglin protein inhibits SNI-induced BSCB dysfunction through TGF-β/Smad2 signaling pathway. Expressions of endoglin, Smad2, p-Smad2, and TGF-βRI in the spinal cord of rats in each group at 3 days after SNI. **(A)** Representative immunoblots. **(B–E)** Data summary (*n* = 3 rats for each group). **(F)** Intrathecal administration of siRNA (Smad2) decreased Smad2 expression in spinal cord (*n* = 3 rats for each group). **(G)** The blood–spinal barrier structure of rats in each group. **(H)** Changes of blood–spinal barrier permeability in each group. **(I)** Quantitative analysis of Evens Blue in the L4–L6 spinal cord of each group. Significance was assessed by two-tailed unpaired Student’s *t*-test **(F)** and one-way ANOVA followed by Tukey’s multiple comparisons test **(B–E)**. ^*^p < 0.05, ^**^p < 0.01, and ^***^p < 0.001. Data were presented as mean ± SEM.

Next, we investigated the mechanism by which intrathecal injection of recombinant endoglin protein inhibits SNI-induced BSCB dysfunction. The TGF-β/Smad signaling pathway plays a pivotal role in regulating the proliferation, migration, and differentiation of vascular endothelial cells, which are critical for maintaining the integrity of the vascular endothelial basement membrane (Massagué and Sheppard, 2023). Recent studies have shown that endoglin can form a signaling complex with TGF-β receptors, leading to the phosphorylation and activation of the downstream Smad signaling pathway (Xueqin et al., 2023). Based on these findings, we hypothesized that intrathecal administration of recombinant endoglin protein may alleviate SNI-induced BSCB dysfunction by activating TGF-β/Smad2 signaling pathway. To test this hypothesis, we assessed the expression of Type I TGF-β receptor (TGF-βRI), Smad2, and phosphorylated Smad2 (p-Smad2) in the L4–L6 dorsal horn at 3 days after SNI. Our results found that SNI significantly decreased the expression of TGF-βRI and the phosphorylation of Smad2 in the spinal cord, while the total levels of Smad2 remained unaltered. Conversely, intrathecal administration of recombinant endoglin protein reversed SNI-induced reduction in both TGF-βRI expression and Smad2 phosphorylation (Figures 8A–E).

To further elucidate the role of the TGF-β/Smad2 signaling pathway in this process, we performed Smad2 knockdown in the spinal cord via intrathecal administration of siRNA (Smad2) and verified the knockdown efficiency using western blot analysis (Figure 8F). Subsequently, we employed transmission electron microscopy to assess the structural and functional integrity of the BSCB in the L4–L6 spinal cord region. The results showed that intrathecal administration of recombinant endoglin protein reversed SNI-induced BSCB dysfunction, but this protective effect of recombinant endoglin protein on BSCB was abolished by knockdown of Smad2. These findings suggest that recombinant endoglin protein alleviates SNI-induced BSCB damage by activating the TGF-β/Smad2 signaling pathway (Figures 8G–I).

### Intrathecal injection of recombinant endoglin protein inhibits SNI-induced NR2B phosphorylation and hyperalgesia via the TGF-β/Smad2 signaling pathway

**Figure 9:**
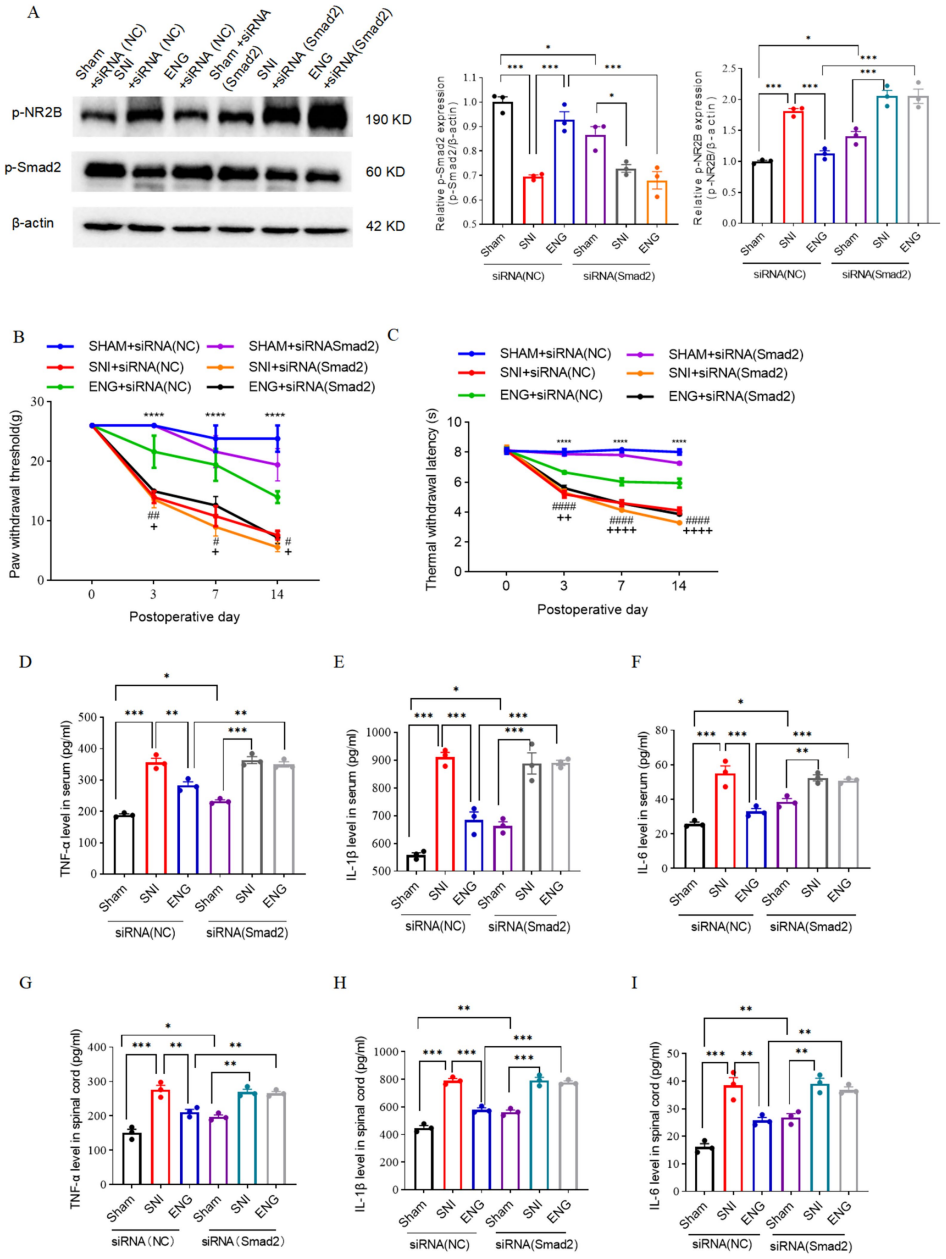
Effect of intrathecal injection of siRNA (Smad2) on SNI-induced NR2B phosphorylation, hyperalgesia, and inflammatory factors in peripheral blood and spinal cord. **(A)** Expressions of p-Smad2 and p-NR2B in the spinal cord of rats in each group at 14 days after SNI; *n* = 3 rats for each group. **(B)** The paw withdrawal threshold of each group of rats on the day of SNI and at 3, 7, and 14 days after SNI; *n* = 5 rats in each group. **(C)** The thermal withdrawal latency of each group of rats on the day of SNI and at 3, 7, and 14 days after SNI. **(D–F)** The ELISA result shows the expression level of TNF-α **(D)**, IL-1β **(E)**, and IL-6 **(F)** in peripheral blood serum 14 days after SNI intervention in each group. The ELISA result shows the expression level of TNF-α **(G)**, IL-1β **(H)**, and IL-6 **(I)** in the spinal cord 14 days after SNI intervention in each group; *n* = 5 rats in each group. Significance was assessed by one-way ANOVA followed by Tukey’s multiple comparisons test **(A)** and two-way repeated-measures ANOVA followed by Tukey’s multiple comparisons test **(B,C)**. ^*^p < 0.05, ^**^p < 0.01, and ^***^p < 0.001. Data was presented as mean ± SEM.

To investigate whether intrathecal injection of recombinant endoglin protein inhibits SNI-induced phosphorylation of NR2B at Tyr1472 and hyperalgesia by activating TGF-β/Smad2 signaling pathway, we performed intrathecal administration of siRNA (Smad2) and recombinant endoglin protein and found that intrathecal administration of recombinant endoglin protein reversed SNI-induced Tyr1472-NR2B phosphorylation, as well as mechanical and thermal hyperalgesia, but this protective effect of recombinant endoglin protein on BSCB was abolished by knockdown of Smad2 (Figures 9A–C). Notably, upon Smad2 knockdown, recombinant endoglin failed to reduce pro-inflammatory cytokine levels in both serum and spinal dorsal horn. Thus, the anti-inflammatory effects induced by endoglin injection were abrogated by Smad2 siRNA interference (Figures 9D–I). These findings suggest that recombinant endoglin protein alleviates SNI-induced NR2B phosphorylation and hyperalgesia by activating TGF-β/Smad2 signaling pathway.

## Discussion

Central neuroinflammation has been recognized as a critical factor in the pathogenesis of neuropathic pain. The onset of central inflammation is often associated with the infiltration of peripheral immune cells and factors that compromise the integrity of the BSCB (Jin et al., 2021; Chen et al., 2018). However, the molecular mechanisms underlying BSCB dysfunction in neuropathic pain remain poorly understood. In this study, we observed a significant reduction in endoglin expression in the peripheral blood of patients with PHN, as well as in the peripheral blood and L4–L6 spinal cord of SNI rats. Intrathecal injection of recombinant endoglin protein effectively alleviated mechanical and thermal hyperalgesia in SNI rats, and significantly reversed SNI-induced microglial activation, inflammation, BSCB permeability impairment, and phosphorylation of NR2B at Tyr1472 in the spinal cord. Notably, we found that SNI led to a significant decrease in the expression of TGF-βRI and phosphorylation of Smad2 in the spinal cord, which was reversed by intrathecal administration of recombinant endoglin protein. Conversely, intrathecal injection of siRNA (Smad2) abolished these protective effects of recombinant endoglin protein. These findings suggest that reduction of endoglin expression disrupts the structural integrity and function of the BSCB through inhibition of the TGF-β/Smad2 signaling pathway in endothelial cells, thereby leading to microglial activation, inflammation, NR2B phosphorylation in spinal cord neurons, and mechanical and thermal hyperalgesia. Furthermore, intrathecal injection of recombinant endoglin protein effectively alleviated SNI-induced mechanical and thermal hyperalgesia. Our study elucidates a key mechanism underlying neuropathic pain pathogenesis and identifies endoglin as a potential therapeutic target for treating this condition.

### Endoglin is involved in chronic neuropathic pain

Sequencing of peripheral blood from patients with postherpetic neuralgia (PHN) revealed a decreased level of endoglin. Endoglin is highly expressed on activated endothelial cells, and its loss in myeloid cells has been shown to trigger spontaneous inflammation (Meurer and Weiskirchen, 2020). Based on these findings, we hypothesized that endoglin may play a role in modulating inflammation and contribute to the development of chronic neuropathic pain. To test this hypothesis, we conducted animal-level studies and found that endoglin expression was significantly reduced in both the peripheral blood and L4–L6 spinal cord of rats with SNI. It is important to note that the SNI model is not a perfect surrogate for PHN. The current lack of effective animal models recapitulating PHN arises from the strict human tropism of Varicella-Zoster Virus (VZV) infection, which precludes the establishment of productive VZV replication in non-human models (van Oorschot et al., 2021). Consequently, commonly employed models attempting to simulate PHN involve either inoculation with VZV strains in primary human embryonic lung fibroblasts or HSV-1-induced pain models in animals. However, the pain phenotype in these models stems from acute VZV or HSV-1 infection, deviating significantly from the characteristic human PHN progression involving viral latency to reactivation. Furthermore, these models demand more stringent laboratory conditions and infrastructure (Sorel and Messaoudi, 2018; Ku et al., 2004; Maayan et al., 1998). Given these limitations, we selected the SNI model as a surrogate to investigate the underlying mechanisms of neuropathic pain. SNI represents a well-established and valuable tool for studying the mechanisms of neuropathic pain following peripheral nerve trauma. Although PHN and the SNI model exhibit etiological and pathophysiological differences, both conditions are fundamentally characterized as neuropathic pain induced by peripheral nerve injury. Nevertheless, it must be acknowledged that utilizing the SNI model to study PHN carries inherent limitations, and extrapolating findings directly to PHN represents a caveat. The primary shortcoming lies in the fundamental divergence in the etiopathogenesis between the model and the human disease.

Importantly, intrathecal injection of recombinant endoglin effectively alleviated mechanical pain and thermal hyperalgesia in SNI rats. Additionally, we observed a significant increase in the phosphorylation of NR2B at Tyr1472 in the L4–L6 spinal cord at 14 days after SNI. Previous studies have demonstrated that the development and maintenance of neuropathic pain following peripheral nerve injury critically depend on phosphorylation of Tyr1472-NR2B in spinal dorsal horn neurons (Katano et al., 2016). These results suggest that endoglin is involved in the development and maintenance of neuropathic pain following peripheral nerve injury.

### Endoglin inhibits inflammation response in chronic neuropathic pain

Central neuroinflammation plays a crucial role in the development of neuropathic pain. Endoglin is widely involved in the regulation of inflammation in various immune diseases, including rheumatic diseases, hereditary hemorrhagic telangiectasia-1 (HHT-1), and preeclampsia (Venkatesha et al., 2006; Mangoni and Zinellu, 2024; Hawinkels et al., 2010; Rossi et al., 2016). Our findings indicate that endoglin can improve the inflammatory response associated with chronic neuropathic pain. Specifically, intrathecal administration of exogenous endoglin significantly inhibited the activation of microglia and the expression of inflammatory factors in the dorsal horn of the ipsilateral spinal cord induced by SNI, while also reducing peripheral nerve injury-induced hyperalgesia.

### Endoglin relieves BSCB dysfunction following peripheral nerve injury

Peripheral nerve injury (PNI) can lead to structural disruption and increased permeability of the blood–spinal cord barrier (BSCB), facilitating the migration of immune cells from circulation into the spinal parenchyma and releasing inflammatory mediators (Chopra et al., 2022). This, in turn, activates glial cells, promoting neuroinflammation and central sensitization, which are critical for the onset and maintenance of neuropathic pain. Endothelial cells are essential components of the BSCB, and studies have shown that endoglin, as a major membrane-associated glycoprotein on endothelial cells, plays a vital role in regulating their physiological state. We found that the downregulation of endoglin expression peaked on the third day post-PNI, coinciding with the deterioration in BSCB structure and function. Notably, this time point is also associated with increased expression of inflammatory factors and microglial activation in the spinal cord. These results suggest that the reduced expression of endoglin may lead to the infiltration of peripheral immune-related cells and inflammatory mediators into the spinal cord. Furthermore, injection of recombinant endoglin inhibited the increased permeability of the BSCB induced by SNI, alleviating central nervous inflammation, mechanical pain, and thermal hyperalgesia in rats. This indicates that decreased expression of endoglin may compromise the normal barrier function of endothelial cells, facilitating the infiltration of peripheral inflammation-related substances into the spinal cord and triggering hyperalgesic responses. Additionally, the third day post-PNI may represent a key time point for endoglin’s involvement in the modulation of neuropathic pain.

### Endoglin relieves BSCB dysfunction through TGF-β/Smad2 signaling pathway

The molecular and cellular mechanisms by which intrathecal injection of endoglin relieves BSCB dysfunction remain unclear. Regulation and redistribution of tight junction (TJ) proteins are considered to be key factors in peripheral nerve injury-induced increased permeability of BSCB (Reinhold and Rittner, 2017; Otani and Furuse, 2020; Ballabh et al., 2004; Ronaldson et al., 2009). Exogenous TGF-βI has been shown to rescue the loss of TJ proteins, thereby restoring the integrity of BSCB (Deng et al., 2024). Endoglin functions as an important membrane-associated protein in the TGF-βI signaling pathway, modulating endothelial cells’ responsiveness to TGF-β signals (Massagué and Sheppard, 2023; Echeverry et al., 2011). Smad proteins serve as critical downstream molecules in TGF-β signaling, relaying signals via TGF-β receptors I and II (TGF-βRI and TGF-βRII) (Xu et al., 2016; Xie et al., 2020). In this study, we found that SNI induced a reduction in the expression of p-Smad2 and TGF-βRI in the spinal cord. Notably, intrathecal administration of recombinant endoglin protein reversed SNI-induced BSCB dysfunction, but this protective effect of recombinant endoglin protein on BSCB was abolished by knockdown of Smad2. These results suggest that TGF-β/Smad2 may be a key signaling pathway for endoglin to regulate the structural integrity and function of the blood–spinal cord barrier, and that intrathecal injection of recombinant endoglin protein alleviates SNI-induced BSCB damage by activating the TGF-β/Smad2 signaling pathway.

Notably, the therapeutic effect observed in this study was evaluated exclusively in a single rat model of neuropathic pain and remains unvalidated in chronic pain models or other species. Furthermore, the analgesic efficacy of endoglin was assessed using only a single dose (2 μg/mL/day, 10 μL; equivalent to 20 g/day). While this dose significantly alleviated neuropathic pain behavior, a comprehensive dose–response relationship remains undefined. Future research should conduct systematic dose–response evaluations to establish the optimal therapeutic window, efficacy gradient, and potential toxicity threshold of endoglin. Although this limitation does not invalidate the conclusions regarding endoglin’s mechanism of action via the TGF-β/Smad pathway, it underscores the necessity for further pharmacological validation of its therapeutic potential. The 14-day treatment period in this study provided preliminary evidence supporting endoglin’s efficacy. Given that human PHN is a chronic condition persisting for months to years, future investigations should incorporate long-term endoglin administration (>28 days), neurotoxicity assessments (histological examination of spinal cord and dorsal root ganglia), and cross-species pharmacokinetic studies. Consequently, confirming endoglin’s clinical translation potential will require multidose gradient experiments, extended treatment durations (>14 days), and validation across multiple models.

**Figure 10:**
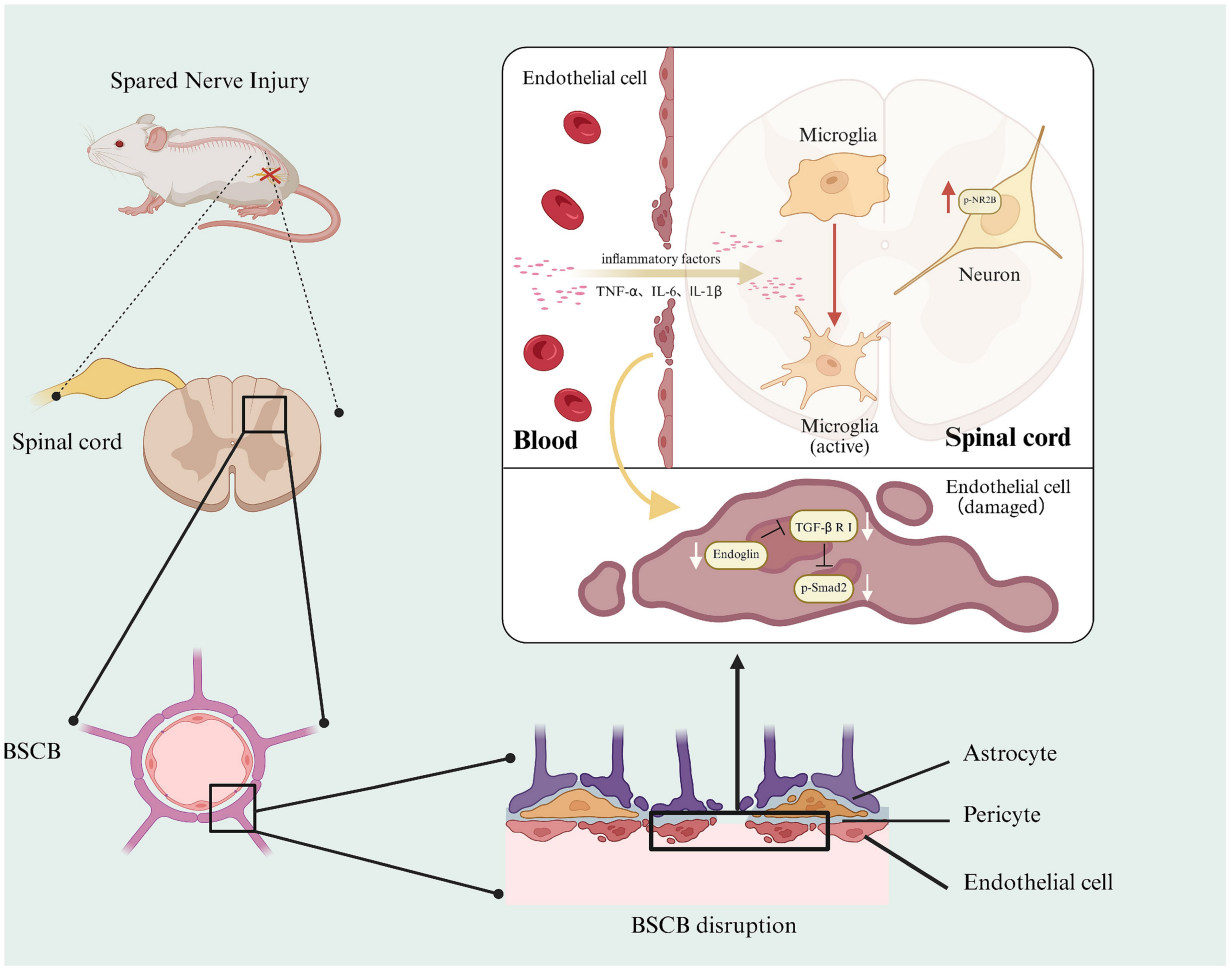
Endoglin regulates the function and structural integrity of BSCB through the TGF-β/Smad2 signaling pathway and is involved in neuropathic pain.

In conclusion, our research suggests that the reduction of endoglin expression disrupts the structural integrity and function of the BSCB through inhibition of the TGF-β/Smad2 signaling pathway in endothelial cells, thereby leading to microglial activation, inflammation, NR2B phosphorylation in spinal cord neurons, and mechanical and thermal hyperalgesia. This study elucidates a key mechanism underlying the pathogenesis of neuropathic pain and identifies a potential therapeutic target for its treatment (Figure 10).

## Data Availability

The original contributions presented in the study are publicly available. This data can be found here: http://www.ebi.ac.uk/pride with project accession PXD067890, token Js2bo2qfxjzs.

## Footnotes

^1^ http://geneontology.org/

^2^ https://www.kegg.jp/

## References

[ref1] Ballabh P. Braun A. Nedergaard M. (2004). The blood-brain barrier: an overview: structure, regulation, and clinical implications. Neurobiol. Dis. 16, 1–13. doi: 10.1016/j.nbd.2003.12.016, PMID: 15207256

[ref2] Basbaum A. I. Bautista D. M. Scherrer G. Julius D. (2009). Cellular and molecular mechanisms of pain. Cell 139, 267–284. doi: 10.1016/j.cell.2009.09.028, PMID: 19837031 PMC2852643

[ref3] Chen B. S. Roche K. W. (2007). Regulation of NMDA receptors by phosphorylation. Neuropharmacology 53, 362–368. doi: 10.1016/j.neuropharm.2007.05.018, PMID: 17644144 PMC2001266

[ref4] Chen G. Zhang Y. Q. Qadri Y. J. Serhan C. N. Ji R. R. (2018). Microglia in pain: detrimental and protective roles in pathogenesis and resolution of pain. Neuron 100, 1292–1311. doi: 10.1016/j.neuron.2018.11.009, PMID: 30571942 PMC6312407

[ref5] Chopra N. Menounos S. Choi J. P. Hansbro P. M. Diwan A. D. Das A. (2022). Blood–spinal cord barrier: its role in spinal disorders and emerging therapeutic strategies. NeuroSci 3, 1–27. doi: 10.3390/neurosci3010001, PMID: 39484675 PMC11523733

[ref6] Cohen S. P. Vase L. Hooten W. M. (2021). Chronic pain: an update on burden, best practices, and new advances. Lancet 397, 2082–2097. doi: 10.1016/S0140-6736(21)00393-7, PMID: 34062143

[ref7] Costigan M. Moss A. Latremoliere A. Johnston C. Verma-Gandhu M. Herbert T. A.. (2009). T-cell infiltration and signaling in the adult dorsal spinal cord is a major contributor to neuropathic pain-like hypersensitivity. J. Neurosci. 29, 14415–14422. doi: 10.1523/JNEUROSCI.4569-09.2009, PMID: 19923276 PMC2813708

[ref8] Costigan M. Scholz J. Woolf C. J. (2009). Neuropathic pain: a maladaptive response of the nervous system to damage. Annu. Rev. Neurosci. 32, 1–32. doi: 10.1146/annurev.neuro.051508.135531, PMID: 19400724 PMC2768555

[ref9] Deng Z. Fan T. Xiao C. Tian H. Zheng Y. Li C.. (2024). TGF-β signaling in health, disease, and therapeutics. Signal Transduct. Target. Ther. 9:61. doi: 10.1038/s41392-024-01764-w, PMID: 38514615 PMC10958066

[ref10] Echeverry S. Shi X. Q. Rivest S. Zhang J. (2011). Peripheral nerve injury alters blood–spinal cord barrier functional and molecular integrity through a selective inflammatory pathway. J. Neurosci. 31, 10819–10828. doi: 10.1523/JNEUROSCI.1642-11.2011, PMID: 21795534 PMC6623085

[ref11] Gao Y. J. Zhang L. Samad O. A. Suter M. R. Yasuhiko K. Xu Z. Z.. (2009). JNK-induced MCP-1 production in spinal cord astrocytes contributes to central sensitization and neuropathic pain. J. Neurosci. 29, 4096–4108. doi: 10.1523/JNEUROSCI.3623-08.2009, PMID: 19339605 PMC2682921

[ref12] Goldim M. P. S. Della Giustina A. Petronilho F. (2019). Using Evans blue dye to determine blood-brain barrier integrity in rodents. Curr. Protoc. Immunol. 126:e83. doi: 10.1002/cpim.83, PMID: 31483106

[ref13] Hawinkels L. J. Kuiper P. Wiercinska E. Verspaget H. W. Liu Z. Pardali E.. (2010). Matrix metalloproteinase-14 (MT1-MMP)-mediated endoglin shedding inhibits tumor angiogenesis. Cancer Res. 70, 4141–4150. doi: 10.1158/0008-5472.CAN-09-4466 20424116

[ref14] Inoue K. Tsuda M. (2018). Microglia in neuropathic pain: cellular and molecular mechanisms and therapeutic potential. Nat. Rev. Neurosci. 19, 138–152. doi: 10.1038/nrn.2018.2, PMID: 29416128

[ref15] Jin L. Y. Li J. Wang K. F. Xia W. W. Zhu Z. Q. Wang C. R.. (2021). Blood–spinal cord barrier in spinal cord injury: a review. J. Neurotrauma 38, 1203–1224. doi: 10.1089/neu.2020.7413, PMID: 33292072

[ref16] Katano T. Fukuda M. Furue H. Yamazaki M. Abe M. Watanabe M.. (2016). Involvement of brain-enriched guanylate kinase-associated protein (BEGAIN) in chronic pain after peripheral nerve injury. eNeuro 3:ENEURO.0110-16.2016. doi: 10.1523/ENEURO.0110-16.2016, PMID: 27785460 PMC5066261

[ref17] Ku C. C. Zerboni L. Ito H. Graham B. S. Wallace M. Arvin A. M. (2004). Varicella-zoster virus transfer to skin by T cells and modulation of viral replication by epidermal cell interferon-alpha. J. Exp. Med. 200, 917–925. doi: 10.1084/jem.20040634, PMID: 15452178 PMC2213285

[ref18] Lertkiatmongkol P. Liao D. Mei H. Hu Y. Newman P. J. (2016). Endothelial functions of platelet/endothelial cell adhesion molecule-1 (CD31). Curr. Opin. Hematol. 23, 253–259. doi: 10.1097/MOH.0000000000000239, PMID: 27055047 PMC4986701

[ref19] Maayan C. Nimrod A. Morag A. Becker Y. (1998). Herpes simplex virus-1 and varicella virus infections in familial dysautonomia patients. J. Med. Virol. 54, 158–161. doi: 10.1002/(SICI)1096-9071(199803)54:3<158::AID-JMV2>3.0.CO;2-4, PMID: 9515762

[ref20] Mangoni A. A. Zinellu A. (2024). A systematic review and meta-analysis of the endothelial-immune candidate biomarker endoglin in rheumatic diseases. Clin. Exp. Med. 25:4. doi: 10.1007/s10238-024-01519-5, PMID: 39535678 PMC11561007

[ref21] Massagué J. Sheppard D. (2023). TGF-β signaling in health and disease. Cell 186, 4007–4037. doi: 10.1016/j.cell.2023.07.036, PMID: 37714133 PMC10772989

[ref22] Meurer S. K. Weiskirchen R. (2020). Endoglin: an ‘accessory’ receptor regulating blood cell development and inflammation. Int. J. Mol. Sci. 21:9247. doi: 10.3390/ijms21239247, PMID: 33287465 PMC7729465

[ref23] Montague-Cardoso K. Malcangio M. (2021). Changes in blood–spinal cord barrier permeability and neuroimmune interactions in the underlying mechanisms of chronic pain. Pain Rep. 6:e879. doi: 10.1097/PR9.0000000000000879, PMID: 33981925 PMC8108584

[ref24] Otani T. Furuse M. (2020). Tight junction structure and function revisited. Trends Cell Biol. 30, 805–817. doi: 10.1016/j.tcb.2020.08.004, PMID: 32891490

[ref25] Peterson C. D. Kitto K. F. Verma H. Pflepsen K. Delpire E. Wilcox G. L.. (2021). Agmatine requires GluN2B-containing NMDA receptors to inhibit the development of neuropathic pain. Mol. Pain 17:17448069211029171. doi: 10.1177/17448069211029171, PMID: 34210178 PMC8255568

[ref26] Rakocevic J. Orlic D. Mitrovic-Ajtic O. Tomasevic M. Dobric M. Zlatic N.. (2017). Endothelial cell markers from clinician's perspective. Exp. Mol. Pathol. 102, 303–313. doi: 10.1016/j.yexmp.2017.02.005, PMID: 28192087

[ref27] Reinhold A. K. Rittner H. L. (2017). Barrier function in the peripheral and central nervous system-a review. Pflugers Arch. 469, 123–134. doi: 10.1007/s00424-016-1920-8, PMID: 27957611

[ref28] Ronaldson P. T. Demarco K. M. Sanchez-Covarrubias L. Solinsky C. M. Davis T. P. (2009). Transforming growth factor-beta signaling alters substrate permeability and tight junction protein expression at the blood-brain barrier during inflammatory pain. J. Cereb. Blood Flow Metab. 29, 1084–1098. doi: 10.1038/jcbfm.2009.32, PMID: 19319146 PMC3910515

[ref29] Rossi E. Smadja D. M. Boscolo E. Langa C. Arevalo M. A. Pericacho M.. (2016). Endoglin regulates mural cell adhesion in the circulatory system. Cell. Mol. Life Sci. 73, 1715–1739. doi: 10.1007/s00018-015-2099-4, PMID: 26646071 PMC4805714

[ref30] Sauer R. S. Kirchner J. Yang S. Hu L. Leinders M. Sommer C.. (2017). Blood–spinal cord barrier breakdown and pericyte deficiency in peripheral neuropathy. Ann. N. Y. Acad. Sci. 1405, 71–88. doi: 10.1111/nyas.13436, PMID: 28753236

[ref31] Shasha T. Gruijs M. van Egmond M. (2022). Mechanisms of colorectal liver metastasis development. Cell. Mol. Life Sci. 79:607. doi: 10.1007/s00018-022-04630-6, PMID: 36436127 PMC9701652

[ref32] Sorel O. Messaoudi I. (2018). Varicella virus-host interactions during latency and reactivation: lessons from simian varicella virus. Front. Microbiol. 9:3170. doi: 10.3389/fmicb.2018.03170, PMID: 30619226 PMC6308120

[ref33] Tenorio G. Kulkarni A. Kerr B. J. (2013). Resident glial cell activation in response to perispinal inflammation leads to acute changes in nociceptive sensitivity: implications for the generation of neuropathic pain. Pain 154, 71–81. doi: 10.1016/j.pain.2012.09.008, PMID: 23103436

[ref34] van Oorschot D. Vroling H. Bunge E. Diaz-Decaro J. Curran D. Yawn B. (2021). A systematic literature review of herpes zoster incidence worldwide. Hum. Vaccin. Immunother. 17, 1714–1732. doi: 10.1080/21645515.2020.1847582, PMID: 33651654 PMC8115759

[ref35] Venkatesha S. Toporsian M. Lam C. Hanai J. Mammoto T. Kim Y. M.. (2006). Soluble endoglin contributes to the pathogenesis of preeclampsia. Nat. Med. 12, 642–649. doi: 10.1038/nm1429, PMID: 16751767

[ref36] Xie H. Chen Y. Du K. Wu W. Feng X. (2020). Puerarin alleviates vincristine-induced neuropathic pain and neuroinflammation via inhibition of nuclear factor-κB and activation of the TGF-β/Smad pathway in rats. Int. Immunopharmacol. 89:107060. doi: 10.1016/j.intimp.2020.107060, PMID: 33049496

[ref37] Xu J. Li P. Lu F. Chen Y. Guo Q. Yang Y. (2023). Domino reaction of neurovascular unit in neuropathic pain after spinal cord injury. Exp. Neurol. 359:114273. doi: 10.1016/j.expneurol.2022.114273, PMID: 36375510

[ref38] Xu F. Liu C. Zhou D. Zhang L. (2016). TGF-β/SMAD pathway and its regulation in hepatic fibrosis. J. Histochem. Cytochem. 64, 157–167. doi: 10.1369/0022155415627681, PMID: 26747705 PMC4810800

[ref39] Xueqin C. Jingyue Y. Huan L. Lei L. Xupin L. Bo L.. (2023). Exploring the mechanism of Buyang Huanwu decoction alleviating restenosis by regulating VSMC phenotype switching and proliferation by network pharmacology and molecular docking. Curr. Comput. Aided Drug Des. 19, 451–464. doi: 10.2174/1573409919666230203144207 36740793

[ref40] Zhang J. Shi X. Q. Echeverry S. Mogil J. S. De Koninck Y. Rivest S. (2007). Expression of CCR2 in both resident and bone marrow-derived microglia plays a critical role in neuropathic pain. J. Neurosci. 27, 12396–12406. doi: 10.1523/JNEUROSCI.3016-07.2007, PMID: 17989304 PMC6673247

