# Correction: Endoglin alleviates neuropathic pain by protecting the blood–spinal cord barrier through the TGF-β/Smad2 signaling pathway

**DOI:** 10.3389/fnmol.2025.1707410

**Published:** 2025-09-29

**Authors:** SiJing Liao, Fei-Ran Zhou, Qun Li, Tao Jin, Gui-Fang Xiang, Xin Wang, Yan Liu, Hq Zhu, Qing Liu, Yuexin Liu, Ying Zhang

**Affiliations:** ^1^Traditional Chinese Medicine Hospital of Meishan, Meishan, China; ^2^The Affiliated Hospital of Southwest Medical University, Luzhou, China; ^3^The Affiliated Traditional Chinese Medicine Hospital, Southwest Medical University, Luzhou, China; ^4^Suining First People's Hospital, Suining, China; ^5^Hejiang Traditional Chinese Medicine Hospital, Luzhou, China; ^6^Zhongshan Hospital, Fudan University, Shanghai, China; ^7^Luzhou Key Laboratory of Research for Integrative on Pain and Perioperative Organ Protection, Luzhou, China

**Keywords:** endoglin, TGF-β/Smad2, neuropathic pain, blood–spinal cord barrier, endothelial cell

Affiliation 1. Traditional Chinese Medicine Hospital of Meishan, Meishan, China was erroneously given as 1. Traditional Chinese Medicine Hospital of Meishan, Meisham, China.

Affiliation 3. The Affiliated Traditional Chinese Medicine Hospital, Southwest Medical University, Luzhou, China was erroneously given as 3. Affiliated Traditional Chinese Medicine Hospital, Southwest Medical University, Luzhou, China.

Affiliation 5. Hejiang Traditional Chinese Medicine Hospital, Luzhou, China was erroneously given as 5. Hejiang County Traditional Chinese Medicine Hospital, Luzhou, China.

The original version of this article has been updated.

